# Child-oriented marketing techniques in snack food packages in Guatemala

**DOI:** 10.1186/1471-2458-13-967

**Published:** 2013-10-18

**Authors:** Violeta Chacon, Paola Letona, Joaquin Barnoya

**Affiliations:** 1 Department of Research, Cardiovascular Surgery Unit of Guatemala, Guatemala City, Guatemala; 2 Division of Public Health Sciences, Department of Surgery, Washington University in St. Louis, School of Medicine, St. Louis, Missouri, USA

**Keywords:** Marketing, Snack food, Children

## Abstract

**Background:**

Childhood overweight in Guatemala is now becoming a public health concern. Child-oriented marketing contributes to increase children’s food preference, purchase and consumption. This study sought to assess the availability of child-oriented snack foods sold in school kiosks and convenience stores near public schools in Guatemala, to identify the marketing techniques used in child-oriented snack food packages and to classify the snacks as “healthy” or “less-healthy”.

**Methods:**

We purchased all child-oriented snacks found in stores inside and within 200 square meters from four schools in an urban community. Snacks were classified as child-oriented if the package had any promotional characters, premium offers, children′s television/movie tie-ins, sports references, or the word “child”. We used a checklist to assess child-oriented references and price. Snacks were classified as “healthy” or “less-healthy” according to the UK standards for the Nutritional Profiling Model.

**Results:**

We analyzed 106 packages found in 55 stores. The most commonly used technique was promotional characters (92.5%) of which 32.7% were brand-specific characters. Premium offers were found in 34% of packages and were mostly collectibles (50%). Most marketing techniques were located on the front and covered nearly 25% of the package surface. Median (interquartile range) price was US$ 0.19 (0.25). Nutrition labels were found in 91 (86%) packages and 41% had a nutrition related health claim. Most snacks (97.1%) were classified as “less-healthy”.

**Conclusion:**

In Guatemala, the food industry targets children through several marketing techniques promoting inexpensive and unhealthy snacks in the school environment. Evidence-based policies restricting the use of promotional characters in unhealthy snack food packages need to be explored as a contributing strategy to control the obesity epidemic.

## Background

Childhood obesity is a worldwide public health concern. Nearly 35 million obese children less than 5 years of age live in low-middle income countries (LMIC) [1]. Guatemala, a LMIC, is currently struggling with the double burden of disease [2], where perinatal and infectious diseases coexist with chronic non-communicable diseases as a result of demographic and lifestyle changes. These changes include an aging population coupled with the adoption of unhealthy diets and sedentary lifestyles [3,4]. While nutritional stunting is still highly prevalent (45.6%) [5], overweight is now becoming a public health concern (32.6% and 32.5% in public and private school-age children, respectively) [6].

Food marketing influences preference, stimulates demand, increases purchase frequency, builds brand awareness and loyalty, and encourages children to try new products [7]. Marketing techniques (e.g. packaging, product design and placement) effectively create brand recognition at the point-of-sale among children as young as 2 to 3 years of age [7]. Consequently, children persuade parents to purchase child-oriented snack foods through pester power [8,9]. Child-oriented packaging with brand-specific or licensed characters from popular movies and television programs is designed to attract children’s attention. Food branding and licensed characters on packaging have been found to significantly influence children’s snack preferences [10,11]. Given that they are used for high sugar, fat and sodium foods [12], these marketing techniques contribute, in part, to increase food preference, purchase and consumption of these foods [13-15], and therefore lead to an increased risk of obesity.

In Guatemala, nutrition labeling/labels is required in all packaged foods and is regulated by the Food Control and Regulation Department of the Ministry of Health according to the Central American Technical Regulation [16]. The consistency of the nutrition health claims with the nutrition information in the label is also regulated. However, as of July 2013, there is no regulation on marketing to children.

Schools in Guatemala have food kiosks that are owned by independent food vendors and sell food snacks and beverages. Therefore, this study sought to assess the availability of child-oriented snack foods sold in school kiosks and convenience stores near public schools in Guatemala, to identify the marketing techniques used in child-oriented snack food packages, and to classify the child-oriented snacks as “healthy” or “less-healthy”.

## Methods

We conveniently selected four (out of 95) public schools (two preschool and two elementary) located in the Municipality of Mixco. Mixco is the third largest city in the Guatemala Department and has a population of 483,705 inhabitants [17]. Permission was obtained from the School District Supervisor and each school’s principal to visit the food store inside each school. We also surveyed all stores located within a circle of area 200 m^2^ centered on the school’s entrance. This distance was measured from the school entrance using Google™ Earth and chosen considering that public schools lack bus services and therefore most caregivers walk their children to and from school.

In each store, we counted all snacks and child-oriented snack foods. A snack was considered child-oriented if the package had any of the following: promotional characters (i.e., licensed, brand-specific or sports character, cartoon, animal/creature, celebrity), premium offer (i.e., collectible, raffle), children’s television or movie tie-ins, sports references (i.e., soccer ball, team logo), or the word “child” or synonym (e.g., junior). A snack is defined as any ready-to-eat food item that comes in a single-serving package [18,19]. To be consistent and to allow comparisons with what has been previously published in the United States [20], snack foods were classified into nine categories: savory snacks, pastries and cookies, sweetened beverages (i.e., fruit drinks, energy drinks, sports drinks), soft drinks, dairy products, cereals, ice cream and frozen desserts, light soft drinks, fruit and vegetable snacks, or water. Each snack was purchased the first time it was found in a store. If the same snack was found in subsequent stores it was only counted but not purchased.

Packages were coded using a checklist adapted from Bragg, et al. [20], translated to Spanish and then pilot tested in nine snacks purchased in Mixco. The checklist includes brand, price, weight, and nutritional label assessment. It also includes the following marketing strategies: promotional characters, premium offers, children’s television or movie tie-ins (programs or movies targeted to children 4 – 12 years old), sports references, and the word “child” or a synonym. Each package could have one or more of these strategies. Nutrition related health claims (e.g. fortified with vitamins) were also documented. The checklist also assessed the location and size of the child-oriented reference. The checklist was completed after the snacks were purchased. Analysis of price was performed only across the purchased snacks.

To classify snack foods as “healthy” or “less-healthy” we used the Nutrient Profile Model (NPM) [21,22]. The NPM results from subtracting “C” from “A” points. “C” points (healthy nutrients, range from 0 to 15) are calculated by adding the fruits, vegetables and nuts, fiber, and protein contents on a scale from 0 (least) to 5 (most) each. “A” points (less-healthy nutrients, range 0 to 40) result from adding the energy, saturated fat, total sugar, and sodium (“less-healthy nutrients”) content on a scale from 0 (least) to 10 (most) each. Nutrient scores are allocated based on the content of 100 grams of each snack. A score of 4 or more for snacks and 1 or more for drinks is classified as “less-healthy” according to the United Kingdom Department of Health standards for child-oriented food advertisement [22]. If a nutrition label was not available in the package, we looked up the industry’s website or contacted by telephone to ask for the information.

We used REDCap™ web-based application for data entry. Descriptive statistics were used to summarize the food categories and marketing techniques. Mean (standard deviation, SD) or median (25^th^ – 75^th^ percentiles) were used when appropriate. Analyses were done with Kruskal-Wallis (interval variables) and Chi-square (nominal variables) tests using STATA® software (version 11.1, 2009).

## Results

We assessed 55 stores (small and large convenience stores and street vendors) around two preschool (4 to 6 years old students) and two elementary (7 – 12 years old) public schools along with the food kiosks inside each school. The schools are located in a low-income neighborhood in Guatemala City and number of students ranges between 230 and 600.

**Figure 1:**
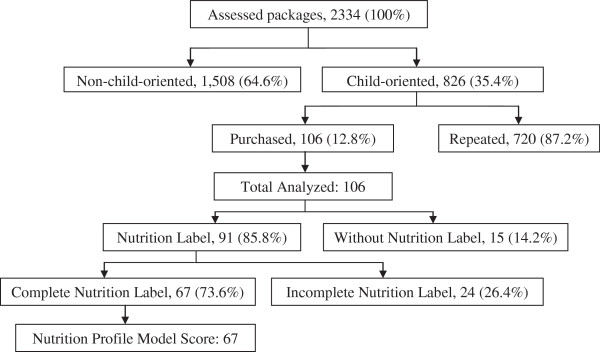
Flow diagram of analyzed snack food packages.

We counted 2334 snack food packages of which 826 (35%) were child-oriented. Of these, 106 were purchased and analyzed (Figure 1). Child-oriented snacks were available in all stores and the most common types were savory snacks, followed by pastries and cookies. There were no child-oriented light soft drinks (Table 1).

**Table 1:** Child-oriented snack food packages by category (n = 2334)

	**Total, n**	Child-oriented, n (%)*
Savory snacks	987	348 (35.3)
Pastries and cookies	592	222 (37.5)
Sweetened beverages	311	93 (29.9)
Cereals	82	61 (74.4)
Soft drinks	266	75 (28.2)
Dairy products	35	15 (42.8)
Ice cream and frozen desserts	25	3 (12.0)
Fruit or water	36	9 (25.0)
Light soft drinks	0	0 (0.0)

*As a percentage of all the packages found in stores.

Median price (25^th^ – 75^th^ percentile) of the snacks was US$ 0.19 (0.12 – 0.38) and ranged from US$ 0.13 (0.09 – 0.19) for savory snacks to US$ 0.44 (0.44 – 0.50) for dairy products (p < 0.05, Table 2).

**Table 2:** Price (US$) of purchased child-oriented snack foods by category (n = 106)

	Median cost (p25 - p75)*
Savory snacks	0.13 (0.09 – 0.19)
Pastries and cookies	0.22 (0.11 – 0.50)
Sweetened beverages	0.32 (0.25 – 0.44)
Cereals	0.13 (0.06 – 0.35)
Soft drinks	0.32 (0.25 – 0.63)
Dairy products	0.44 (0.44 – 0.50)
Ice cream and frozen desserts	0.13 (0.13 – 0.32)
Fruit or water	0.25 (0.13 – 0.50)

*p < 0.05 across food categories.

Regarding marketing techniques, we found promotional characters in most packages (92.5%). Although most were brand-specific characters, we also found cartoon characters and creature/animals (Table 3). Premium offers were also found (36, 34%) and half (18, 50%) were collectibles (Table 3). Most techniques were located in the front and covered nearly 25% of the package surface (Table 4).

**Table 3:** Marketing techniques used in packages (n = 106)*

	**n (%)**	**95% CI**
Promotional characters	98 (92.5)	0.868 – 0.972
Brand-specific character	32 (32.7)	0.235 – 0.418
Cartoon	28 (28.6)	0.194 – 0.378
Animal or creature	26 (26.5)	0.184 – 0.357
Child	15 (15.3)	0.082 – 0.224
Sports personality	8 (8.2)	0.031 – 0.143
Licensed character	7 (7.1)	0.020 – 0.122
Premium offers	36 (34.0)	0.255 – 0.434
Collectible	18 (50.0)	0.333 – 0.667
Game	8 (22.2)	0.083 – 0.361
Extra product	6 (16.7)	0.056 – 0.306
Raffle	3 (8.3)	0.000 – 0.194
Other	2 (5.6)	0.000 – 0.139
Sports references	8 (7.6)	0.028 – 0.132
Television or movie tie-ins	8 (7.6)	0.028 – 0.132
Word “child”	1 (0.9)	0.000 – 0.028

*one package can have more than one marketing technique.

**Table 4:** Location and size of the child-oriented marketing techniques in packages (n=106)

	**n (%)**
Only Front	47 (44.3)
Front and back	47 (44.3)
Front or back, and on one side	4 (3.8)
More than two sides	8 (7.6)
Size	
As 25% of package	68 (64.2)
26 – 50%	29 (27.4)
51 – 75%	6 (5.7)
76 – 100%	3 (2.8)

Nutrition labels/labeling were found in 91 (86%) packages and 41% had nutrition related health claims. Four packages (3.8%) with nutrition related health claims did not have a nutrition label. We then calculated the NPM score of 69 (65.1%) packages (Figure 1). Thirty-seven packages were not analyzed due to incomplete information on one or more nutrients needed to calculate the NPM. For those with an NPM, 67 (97.1%) were classified as “less-healthy”. “Healthy” snacks (2, 2.9%) were two water bottles.

## Discussion

According to our results, in Guatemalan convenience stores near public schools savory snacks are the most frequently found child-oriented snacks. Placing promotional characters on the snack food package is the most frequently used marketing technique to reach children. Furthermore, most child-oriented snacks are classified as unhealthy.

Our findings are consistent with those of child-oriented snack food packages found in supermarkets in the United States and Australia, where most have promotional characters and are classified as unhealthy [23,24]. Promotional characters have been found to influence children’s food choices as they are more likely to choose a snack with a character on the packaging compared to one without a character [10,11]. However, these results were not the same for healthy snacks, such as carrots [10]. In Guatemala, most packages had promotional characters and the most frequent type was brand-specific characters. These characters are created by the food industry with the sole purpose of brand promotion and to increase product recognition by children and parents [14,25]. While the industry has to pay a license fee to use a character, creating its own brand-specific characters appears to be a less expensive option. This might explain, in part, why brand-specific characters were the most frequently found promotional characters on packages. Due to the effects on food preferences and overall nutritional quality [10], restricting the use of child-oriented licensed and brand-specific characters on the packaging of snack foods is needed to discourage consumption of less-healthy snacks.

While low-income families are more likely to be obese, they are also more sensitive to higher food prices [26]. Our results yield that compared to one piece of bread (lowest price US$ 0.04) purchased in the same type of store and neighborhood, savory snacks (mean price US$ 0.19) are on average three times more expensive. Furthermore, the average monthly income in urban Guatemala is US$ 349 compared to US$ 2,326 in the United States [27,28]. Therefore, the motivation to purchase snack foods may not be related to price. This suggests that other strategies (e.g. marketing restrictions), besides taxation [29], might be required to discourage consumption of unhealthy snacks.

Another marketing technique found in our study was the use of premium offers. These have been associated with co-branding in order to market movies, toys and licensed characters in products like soft drinks and breakfast cereals [30]. The food industry promotes several snack purchases by offering different types of toys for children to collect [7]. Efforts to regulate snack food marketing should include restricting or banning the use of toy giveaways to discourage consumption of energy dense snacks.

Other industries have also used characters, promotions, and the package to reach customers. For example in the 1990s the tobacco industry used Joe Camel which had the same impact as Mickey Mouse in reaching pre-school children [31]. Promotions such as buy one pack and get one free and free non-cigarette specialty items attached to the package are also being used by the tobacco industry [6]. These marketing techniques in cigarette packages create brand recognition and loyalty [31]. Moreover, the removal of all promotional techniques from snack food packages and the use of plain packaging may also be an effective strategy to reduce the attractiveness and brand appeal of unhealthy snacks. However, the food industry would likely oppose legislations restricting marketing to children. As with tobacco, the food industry has a strong political lobbying to hold back government action using strategies like diverting attention to other issues (e.g., physical activity) and generating controversy [32].

In Guatemala, nutrition labeling/labels in all food packages is mandatory by law. However, almost 20% of packages were found to be non-compliant. Additionally, of those with a label, one third had incomplete information to classify them as healthy or less-healthy. In addition to enforcing the use of nutrition labeling/labels, health authorities should consider alternative strategies to inform customers. Some alternatives, including traffic lights with caloric intake or front-of-package labeling implemented in the United States and Netherlands [8,32], may be easier to understand and could help children identify healthy snacks.

Health claims can mislead children to perceive snack foods as a healthy product [33]. In Guatemala, nearly half the snacks surveyed had a nutrition related health claim even though most snack foods were classified as “less-healthy”. Enforcing regulation of health claims standards and definitions allowed in snack food packages, similar to those implemented in Australia and New Zealand are required to protect children from misleading claims [16].

Our findings should be considered in light of some limitations. We only purchased snacks that could be seen at the point-of-sale; therefore those out of sight were not included. Additionally, similar to the Bragg, et al. [20] study, confectioneries were not included and therefore our results cannot be generalized to these snacks that are also marketed to children. We only included commercially packaged snacks. Consequently, fruit and vegetables that are not commercially packaged (and most likely do not include any marketing strategies) but can still be consumed as snacks were not included. Furthermore, other package characteristics (e.g. color, shape) that have also been reported as marketing strategies were not assessed [14]. Also, we did not consider online marketing/social media promoted on food packages, another recognized marketing channel [34]. Snack foods sales and availability were not evaluated therefore we were not able to give equal weight to each package in the sample. Finally, even though our sample was not intended to be representative of the entire country, these marketing techniques are likely to be the same nationwide considering snack foods are produced in large scale and most likely are available in every convenience store. Regardless of these limitations, our study is the first one to document unhealthy snack foods advertising strategies targeted to children in an LMIC. Further research is needed to compare marketing techniques in child-oriented and non-child-oriented snack food packages.

## Conclusions

In conclusion, our results yield that promotional characters and premium offers are used by the food industry to promote unhealthy snack foods to Guatemalan children in and around public schools. Further research is warranted to assess the impact these marketing strategies have on unhealthy snack foods consumption and children’s weight over time.

## Competing interests

The authors declare that they have no competing interests.

## Author’s contributions

VC was responsible for the data analysis, interpretation and led the manuscript writing. PL participated in study design and assisted in manuscript writing. JB designed the study, participated in data analysis and interpretation and approved the final version of the manuscript. All authors approved the final version of the manuscript.

## Pre-publication history

The pre-publication history for this paper can be accessed here:

http://www.biomedcentral.com/1471-2458/13/967/prepub
